# Creating Engaging Health Promotion Campaigns on Social Media: Observations and Lessons From Fitbit and Garmin

**DOI:** 10.2196/10911

**Published:** 2018-12-10

**Authors:** Sarah Edney, Svetlana Bogomolova, Jillian Ryan, Tim Olds, Ilea Sanders, Carol Maher

**Affiliations:** 1 Alliance for Research in Exercise, Nutrition and Activity University of South Australia Adelaide Australia; 2 Ehrenberg-Bass Institute for Marketing Science University of South Australia Adelaide Australia; 3 Commonwealth Scientific and Industrial Research Organisation Adelaide Australia

**Keywords:** social media, engagement, physical activity

## Abstract

**Background:**

The popularity and reach of social media make it an ideal delivery platform for interventions targeting health behaviors, such as physical inactivity. Research has identified a dose-response relationship whereby greater engagement and exposure are positively associated with intervention effects, hence enhancing engagement will maximize the potential of these interventions.

**Objective:**

This study examined the social media activity of successful commercial activity tracker brands to understand which creative elements (message content and design) they use in their communication to their audience, which social media platforms attract the most engagement, and which creative elements prompted the most engagement.

**Methods:**

Posts (n=509) made by Fitbit and Garmin on Facebook, Twitter, and Instagram over a 3-month period were coded for the presence of creative elements. User engagement regarding the total number of likes, comments, or shares per post was recorded. Negative binomial regression analyses were used to identify creative elements associated with higher engagement.

**Results:**

Engagement on Instagram was 30-200 times higher than on Facebook, or Twitter. Fitbit and Garmin tended to use different creative elements from one another. A higher engagement was achieved by posts featuring an image of the product, highlighting new product features and with themes of self-improvement (*P*<.01).

**Conclusions:**

Findings suggest that Instagram may be a particularly promising platform for delivering engaging health messaging. Health messages which incorporate inspirational imagery and focus on a tangible product appear to achieve the highest engagement. Fitbit and Garmin employed difference creative elements, which is likely to reflect differences in their target markets. This underscores the importance of market segmentation in health messaging campaigns.

## Introduction

### Background

Social media platforms such as Facebook, Twitter, and Instagram are increasingly becoming a central part of daily life. Today, social media is used for everything from staying in touch with friends and family and accessing news media coverage to keeping up to date with brands and celebrities. Each platform has a large global user base. Facebook has more than two billion active users while Twitter and Instagram have 700 and 328 million each, respectively [1]. Most demographics are well represented on one or more platforms [2], and most aspects of these platforms are free to use. Facebook and Instagram appear to have a relatively equal balance of male and female users, with 50% of Instagram users and 52% of Facebook users being female [3]. Twitter, on the other hand, appears to have more male users (64%) than females (36%). Regarding age demographics, Instagram is more popular among young people with 71% of users being 34 years old or younger, compared to 22% of Facebook users and 40% of Twitter users [3]. It is unsurprising then that these platforms are attracting attention as potential vehicles for the delivery of health promotion and behavior change interventions [4].

To date, most social media-based health behavior intervention and promotion efforts have been based on Facebook or Twitter [4], although they are starting to appear on Instagram [5]. The emerging body of research examining electronic health (eHealth) and mobile health (mHealth) interventions has identified a dose-response relationship, where greater engagement from participants and therefore exposure to an intervention is positively associated with both retention and positive intervention effects [6-8]. However, when subjected to empirical evaluation these interventions often report low rates of engagement which potentially limits their effectiveness [4,9]. The seminal hierarchical behavior change model awareness-interest-desire-action (AIDA), suggests consumer likes, reactions, and responses to commercial posts could serve as proxies for awareness and interest, which are precursors to intentions and eventual joining of health promotion programs and interventions [10]. The potential for such engagement to translate into positive intervention effects highlights a need to understand and enhance user engagement to maximize potential benefits.

The field of engagement science has expanded in recent years. Empirical work undertaken to understand, quantify, and make recommendations to enhance engagement with social media-based health intervention and promotion efforts varies in methodology. Several studies have used subgroup analyses of the intervention arms of randomized controlled trials intervening on weight loss [6] and physical activity [11], within Facebook settings. Content analysis approaches have been used to examine engagement with health-related social media content. For example, Guidry and colleagues [12] used this approach to understand how prominent public health organizations use Twitter and Instagram to disseminate information relating to infectious disease outbreaks, using the 2013-2014 Ebola outbreak as a case study. The authors examined the content of social media posts and the responses (ie, engagement) from users, concluding that Instagram holds particular promise as it elicited significantly higher rates of engagement from users when compared to Twitter.

The study by Rus and Cameron [13] recently examined how health topics are communicated and engaged with in online settings. They analyzed social media posts from diabetes-related Facebook groups to identify which post features elicited different forms of engagement (ie, “likes,” “comments,” or “shares”) from users. Their content analysis approach categorized the post content and then used regression analyses to determine which message features were predictive of engagement. The authors were able to make recommendations for the design of future health-related social media content. Namely, that the use of imagery resulted in a post receiving more “likes” and “shares,” and information relating to the consequences of having diabetes or positive self-identify resulted in more sharing of the post. Whereas posts containing negative affect or social support resulted in more comments.

The popularity of personal health and activity tracker devices (hereafter referred to as wearables) shows little signs of waning [14]. A plethora of wearable brands and models are currently available to consumers at various price points and with a range of features and styles. Two of the largest brands, Fitbit and Garmin, have well established social media profiles across Facebook, Twitter, and Instagram, all with large numbers of real users engaging with their branded content. These commercial wearables may act as a health behavior intervention, although they have less of a focus on determining which of their components are affecting positive behavior change, have far greater resources for software development and marketing, and a primary focus on product sales or revenue [15-17]. Wearables typically contain features that are found in health behavior interventions, for example, self-monitoring capabilities, which are well-established to have a potent influence on changing health behavior [18,19] are often a key feature. Wearables are typically marketed as devices to help users improve their health, and this means they potentially attract the same demographic of the user as health behavior change interventions. While the field of research is in its infancy, there is some emerging evidence to suggest that wearables may be efficacious in changing physical activity behaviors and that they may be able to act as a health intervention [20-25].

The popularity of social media platforms and wearable devices continues to grow while social media-based health interventions continue to report lower than intended rates of engagement [4,9]. Fitbit and Garmin are likely to use particular creative elements in their social media posts. Creative elements seek to translate the content of intended and targeted messages (eg, social media posts) into specific communication elements which include design and content features such as imagery, typeface (ie, within traditional print media), the content of the text, and any interactive features [26]. Examination of the social media activity of commercial wearables and the creative elements used in their posts may provide insights into the type of content that is appealing to current or prospective users of wearables. These insights can then be used to inform the development of appealing content that can be integrated into social media-based health interventions, thereby increasing the appeal and encouraging participants to engage with such interventions. In turn, increased engagement may boost adherence and positive intervention effects [6-8].

### Research Aims

The aims of this study were:

- To examine the social media activity of commercially available wearable activity tracker brands to understand how they engaged social media users
- To determine which platform attracted the most engagement from users
- To examine which creative elements (message content and design elements) elicited higher engagement from users

## Methods

This cross-disciplinary study is a content analysis of publicly-available social media posts made by successful commercial wearable activity trackers brands, Fitbit and Garmin, on their company Facebook, Twitter, and Instagram profiles. Approval for this study was granted by the University of South Australia’s Human Research Ethics Committee (protocol no. 0000036513).

### Sample Selection and Data Collection

Fitbit and Garmin were selected for inclusion based on their 2016 third-quarter worldwide market share figures of 23.0% and 5.7%, respectively and making them the first and third leading wearable activity tracker brands, globally [9]. The brand Xiaomi was second with 16.5% of market share for the same quarter [14]. However, the brand was not included in the sample here as their consumer base is heavily skewed toward a domestic market of Chinese users, the brand has a presence on Facebook and Twitter only and with many posts written in Chinese. Although originally included within the scope of the study, the social media profiles of Jawbone were inactive from early January 2017, and the company has since commenced liquidation [27] and was therefore removed from the analysis here.

All social media posts (n=509) made by the corporate social media accounts of Fitbit and Garmin on Facebook, Twitter, and Instagram over a three-month period (December 2016 to February 2017) were retrospectively collected at the end of March 2017. This three-month period is longer than that used in comparable studies which range from one week through to just under two months [13,28,29]. Posts made on these pages by social media users were not included. A screenshot of each social media post capturing the image, caption, and number of “likes,” “comments,” and “shares” was taken, and assigned an identification number.

Before statistical analysis, the engagement by social media users, regarding the total number of “likes,” “comments,” and “shares” per post was manually extracted from each screenshot and recorded in a Microsoft Excel file. Preliminary exploratory data collection determined that across all three platforms (ie, Facebook, Twitter, and Instagram) almost all user engagement (ie, “likes,” “comments,” and “shares”) with each post occurred within seven days of posting. By 21 days, our exploratory work determined that the amount of engagement had increased by just 2.9% (range 0%-11%) per post, indicating that collecting all engagement data at a single time point would provide an accurate indication of engagement for each post. The number of followers of each brand on each platform at the time of data collection was also recorded.

### Developing the Codebook

A standardized codebook of creative elements was developed to classify the content of the social media posts. Creative elements refer to message content and execution factors used to design communication with the greatest chance of eliciting the desired response from the target audience [30-32] in this case, user engagement. The creative elements that receive the most engagement can then be used in future social media-based health promotion and intervention efforts to aid participant engagement. Development of the codebook was guided by Stewart and Furse [31] who examined the influence of television advertising execution techniques on sales effectiveness. This codebook and study design have been replicated in full [30,32], partially [33,34], and partially for application to examining interactive television advertising [35]. An iterative process was used to condense the original 160-item codebook down to 34 items relevant to social media and health behaviors to accommodate the differences in advertising media. An initial review of the original 160 items identified 101 that were outside of the scope of the current study. For example, items related to “mechanical” advertisement devices such as the length of time until the product was shown were unlikely to be present in a sample comprised mainly of static imagery and text and were removed. Following the initial reduction, the research team piloted the codebook and identified a further 20 items that were unlikely to contribute to addressing the research aims. For example, “demonstration of the product in use” was removed as simply wearing the device in any scenario would constitute a demonstration of use. Following this, the item “nutrition and health” was expanded into 5 items that capture the overall theme of each of the posts (ie, whether the post primarily featured exercise or physical activity, incidental activity, weight loss, food or nutrition, or sleep information). The final version of the codebook with 34 creative elements can be found in Multimedia Appendix 1.

### Applying the Codebook

The screenshots of each social media post were then cropped to display only the image and caption. These screenshots and the associated identification numbers were then entered into an Excel spreadsheet containing columns for each of the codebook categories. Each coder (SE, SB, JR, TO, IS, or CM) received an Excel file containing approximately 170 social media posts. This meant that each of the 509 social media posts was coded for the presence or absence of each of the 26 dichotomous and 8 categorical creative elements, by 2 independent coders. In addition to the Excel file, coders received a codebook containing a description and relevant example of each item, and training in how to administer the codebook. Total percentage pairwise agreement was acceptable, ranging from 82% to 91% for each pair of coders [36]. Disagreements between coders were assessed and resolved by a third independent coder.

### Statistical Analyses

All statistical analyses were conducted using IBM SPSS version 23 [37]. Given that “likes” were the most common form of engagement in our sample and in similar studies (eg, see [13,38]) the authors made the decision to combine the number of “likes,” “comments,” and “shares” per post into “total engagement”, to encompass all interactions with each post within a single metric. Chi-square tests of homogeneity were used to examine differences in use of creative elements between brands. Following a similar methodology to Rus and Cameron [13], the relationships between creative elements and engagement were examined using multivariate regression analyses. Poisson and negative binomial regression models were selected to account for the nonnormal distribution of count data on the dependent variable. Overdispersion of the count data, the Akaike information criterion (AIC) and the Bayesian information criterion (BIC) indicated negative binomial regression as providing the best fit for the data. Creative elements with a significant univariate association (*P*<.25) were included in purposeful selection modeling [39], and those that had a *P*<.1 and changed the main effects by more than 10% were retained in the final models. Intercoder reliability for the 12 items retained for the final model was assessed using the Cohen kappa [40] and ranged from ĸ=.357 to .913 which is considered fair to almost perfect agreement beyond that of chance [41].

## Results

### Engagement by Platform

Fitbit had more followers than Garmin on each of the three platforms. Fitbit and Garmin both had the most followers on Facebook, then Twitter, and then Instagram. Despite the lower numbers of followers, both Fitbit and Garmin received the most engagement on Instagram. Conversely, although both brands have the largest number of followers on Facebook, the platform was the overall worst performer regarding “total engagement” per post. Table 1 shows the number of posts and followers, and mean number of “likes,” “comments,” “shares,” and “total engagement” on each platform and for each brand on each platform.

Instagram posts received the most engagement by far with a mean of 4181.6 (SD 1413.3) “likes” per post, compared to just 47.1 (SD 118.9) and 65.7 (SD 50.3) for Facebook and Twitter, respectively. Instagram posts also received considerably more comments with a mean of 63.1 (SD 68.2) per post, compared to just 5.3 (SD 12.5) for Facebook and 3.0 (SD 9.7) comments per post for Twitter. Twitter posts were more likely to be shared, with a mean of 26.7 (SD 20.9) per post compared to just 3.3 (SD 20.4) per post on Facebook. Instagram does not offer a share function yet, despite this, “total engagement” for Instagram was still considerably higher at a mean of 4244.8 compared to a mean of 55.6 and 95.4 for Facebook and Twitter, respectively.

### Brands’ Use of Creative Elements

Chi-square tests of homogeneity indicated that there were differences in the use of these devices between the 2 brands. Fitbit was more likely to feature females (χ^2^_3_=49.4, *P*<.01) and indoor settings (χ^2^_4_=139.9, *P*<.01) and their posts emphasized social approval (χ^2^_1_=15.7, *P*<.01) and self-improvement (χ^2^_1_=35.2, *P*<.01) while delivering both positive (χ^2^_2_=77.6, *P*<.01) and rational (χ^2^_2_=19.7, *P*<.01) messages about the components or contents (χ^2^_1_=3.8, *P*<.05), or aesthetics (χ^2^_1_=8.1, *P*<.01) of the product. Fitbit was more likely to feature a nonwhite person (χ^2^_2_=19.9, *P*<.01) than Garmin, although for both brands most people in their images were white (84% for each). Unlike Garmin, Fitbit was also found to encompass a full suite of “lifestyle” factors into their posts by often featuring exercise or physical activity (χ^2^_1_=21.5, *P*<.01), incidental activity (χ^2^_1_=26.7, *P*<.01), weight loss (χ^2^_1_=19.4, *P*<.01), food and nutrition (χ^2^_1_=42.1, *P*<.01) and information related to sleep (χ^2^_1_=8.6, *P*<.01).

Garmin was more likely to feature males (χ^2^_3_=49.4, *P*<.01), celebrities (χ^2^_1_=46.2, *P*<.01), children (χ^2^_1_=12.1, *P*<.01), and animals (χ^2^_1_=23.4, *P*<.01). Garmin also featured exciting activities (χ^2^_1_=85.7, *P*<.01), scenic (χ^2^_1_=92.3, *P*<.01), and outdoor wilderness settings (χ^2^_4_=139.9, *P*<.01). Their posts often used rough and rugged themes (χ^2^_1_=86.2, *P*<.01) concerning setting or choice of activity. Garmin also made more emotional appeals (χ^2^_2_=19.7, *P*<.01), featured new or improved product features (χ^2^_1_=11.6, *P*<.01) and mentioned the product in their text (χ^2^_1_=27.7, *P*<.01) more often.

**Table 1:** Number of followers, mean engagement per platform and by platform and brand.

Platform brand		Followers	Posts, n^b^	Likes, mean (SD)	Comments/replies, mean (SD)	Shares/retweets, mean (SD)	Engagement^a^, mean
**Facebook**							
	Fitbit	1,846,974	62	18.3 (14.7)	6.1 (5.8)	0.5 (2.4)	24.9
	Garmin	1,470,340	84	68.4 (153.1)	4.7 (15.7)	5.3 (26.7)	78.4
	Total	—	146	47.1 (118.9)	5.3 (12.5)	3.3 (20.4)	55.6
**Twitter**							
	Fitbit	313,000	156	84.2 (44.3)	3.8 (11.5)	35.6 (17.9)	123.6
	Garmin	130,000	79	29.1 (40.8)	1.4 (3.4)	9.2 (14.2)	39.7
	Total	—	235	65.7 (50.3)	3.0 (9.7)	26.7 (20.9)	95.4
**Instagram**							
	Fitbit	415,581	58	4537.4 (1408.4)	113.4 (72.4)	—	4650.8
	Garmin	260,864	70	3886.9 (1357.6)	21.5 (18.6)	—	3908.3
	Total	—	128	4181.6 (1413.3)	63.1 (68.2)	—	4244.8

^a^Total engagement is the sum of the mean number of “likes,” “comments,” and “shares” per post.

**Figure 1:**
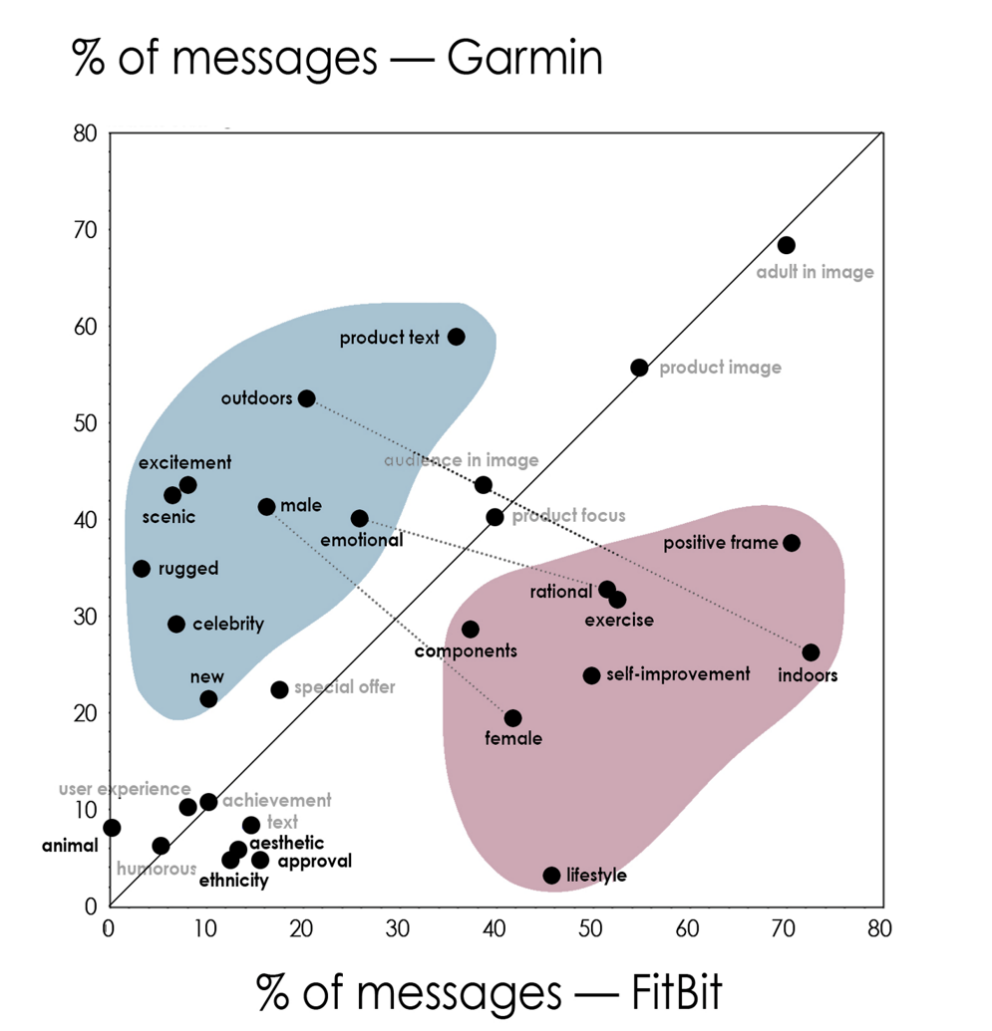
Use of creative elements by Fitbit and Garmin across Facebook, Twitter, and Instagram social media posts. Creative elements appearing in <5% for both Fitbit and Garmin are omitted. Significant differences between Fitbit and Garmin in use of a creative element are included in bold. Creative elements below the identity line are characteristic of Fitbit, while those above the identity line are characteristic of Garmin. The dotted lines connect creative elements that are polar opposites.

Figure 1 illustrates the differences in creative elements used by each brand and the results of chi-square tests of homogeneity that were used to compare differences in frequency of use between brands. Full details of the frequency of use of each creative element by each brand, and the results of chi-square tests of homogeneity to compare differences in frequency of use between brands can be found in Multimedia Appendix 2.

### Creative Elements Associated With Engagement

After controlling for brand and platform, the devices that were most influential on engagement were the mention of new or improved features (*P*<.01), displaying the product in the image (*P*<.01), or themes of self-improvement (*P*<.01). The inclusion of these devices was associated with engagement rates that were 90%, 30%, and 20% higher respectively compared to posts that did not contain these devices. In contrast, engagement rates were found to be between 16% to 45% lower when aesthetic claims (*P*<.01), specific product components or contents (*P*<.01), an outdoor setting (*P*<.01), the mention of a special offer or event (*P*<.01), having text overlaying an image (*P*<.01), using close-up images (*P*<.01), or mentioning a user’s experience (*P*<.05) of the wearable were present in the post, when compared to posts that did not contain these devices. Table 2 presents the results of the multivariate negative binomial regression analyses of creative elements as predictors of “total engagement.”

**Table 2:** Multivariate negative binomial regression analyses of creative elements as predictors of “total engagement.”

Creative elements^a^				B^b^		SE	*P* value	IRR^c^
Intercept^a^				4.371		.117	.000	79.107
New or improved				0.618		.094	.000	1.856
Product in image				0.238		.075	.001	1.269
Self-improvement				0.179		.070	.010	1.196
Aesthetic claims				–0.441		.115	.000	0.643
Components or contents				–0.595		.074	.000	0.552
**Setting^d^**								
		Outdoor wilderness		–0.378		.108	.000	0.685
		Outdoor nature		–0.469		.121	.000	0.626
		Outdoor cityscape		–0.175		.105	.096	0.840
		Indoor setting		–0.109		.092	.234	0.897
		Special offer or event		–0.247		.079	.002	0.781
		Text over image		–0.312		.100	.002	0.732
**Audience in image^e^**								
	Close up image		–0.261		.081		.001	0.770
	Image view through own eyes		0.033		.100		.745	1.033
	User experience		–0.242		.106		.022	0.785
	Children present		–0.332		.218		.128	0.718
	Exercise or physical activity		0.111		.068		.104	1.118

^a^Platform (Facebook, Twitter, and Instagram) included in the final model as a covariate, only creative elements with a *P*<.1 and that changed the main effects by 10% were retained in the final model.

^b^B: beta coefficient.

^c^IRR: incidence rate ratio (is the exponentiation of the regression coefficient, which equates to the odds ratio).

^d^Reference category: no setting.

^e^Reference category: audience not present.

## Discussion

### Principal Findings

This study examined the social media activity of leading brands of wearable activity trackers to make platform and content recommendations for future social media-based health promotion and intervention efforts. The study found that while wearables attracted their largest following on Facebook compared with Twitter or Instagram, engagement with posts was markedly higher on Instagram. Differences in the types of creative elements used were apparent between Fitbit and Garmin. In particular, Fitbit posts were characterized by featuring females and having an upbeat or lighthearted tone whereas Garmin posts featured men, with adventurous and outdoorsy themes. Featuring themes of self-improvement, new product features, or the product in the image were each associated with higher rates of engagement when compared to posts that did not contain these creative elements.

Both Fitbit and Garmin attracted the greatest number of followers on Facebook, which is the largest platform with more than two billion active users, compared to 700 and 328 million for Twitter and Instagram, respectively [1]. However, despite the smaller number of users and followers, it was Instagram that attracted rates of engagement that were from 30 to almost 200 times higher. This is consistent with previous research examining Instagram, where a study comparing dissemination of disease-outbreak information on Twitter and Instagram found higher rates of engagement on Instagram [12]. Furthermore, outside the health research domain, market research has suggested brand advertising receives better audience engagement on Instagram compared with Facebook [42]. Instagram typically has a younger and female audience known to be some of the most prolific social media users [43], and this may also contribute to the higher rates of engagement. It is also possible that engagement is influenced by differences between social media platforms and their respective complex predictive algorithms that control the percentage of followers who view a post, which we were not able to account for in this study.

Creative elements that were associated with the highest engagement were themes of self-improvement, highlighting “new” products or features, and featuring the wearable device in the image. In general, these findings are consistent with previous literature. For example, the scoping study by Van Kessel et al [44] that focused on the development of a social media physical activity intervention for adolescent girls, found that the girls reported a desire for the content of an inspirational, self-improvement nature. Similarly, inspirational imagery taken from Instagram has been found to have a positive effect on motivation to pursue healthy goals in young women, although with the caveat that this needs to be balanced against the potential for Instagram to have a negative effect on body image [45]. In commercial marketing practice, featuring “new” and “improved” products are very common creative elements. Evidence has shown that consumers pay more attention and respond more positively to an advertisement with the word “new” [46] tapping into the novelty effect. As for featuring the product in the image, direct evidence for the effectiveness of such creative elements is mixed [31,32]. However, there is evidence to suggest that featuring the product aids brand awareness and recall [47]. Findings of the current study support this, suggesting that featuring the device is the more effective approach for social media posts. This is likely to be because social media posts need to stand out against the cluttered background, where users are presented with large volumes of posts vying for their attention [48].

The clear differences between how Fitbit and Garmin presented and promoted their products on social media are likely to reflect market segmentation and differences in how Fitbit and Garmin are positioning their respective products. Principles of market segmentation (eg, [32]) have been integrated with social marketing efforts over the past decade [49]. Health promotion and intervention efforts are increasingly mirroring this targeted approach with increasing interest in the use of tailored eHealth and mHealth interventions in recent years and an associated expanding body of evidence suggesting that this tailoring may increase their effectiveness in changing behavior [50-53].

### Strengths and Limitations

This study considered three social media platforms and took a rigorous duplicate coding approach, both of which serve to strengthen the findings. A similar methodology to that presented in the Rus and Cameron [13] analysis of Facebook-based diabetes support groups is followed here, and both studies seek to extend the emerging field of research examining user engagement in order to increase the efficacy of social media-based health interventions and communication. A limitation of the current study was that engagement was operationalized in simple count terms (ie, no. of “likes,” “comments,” and “shares”). The terms “likes,” “comments,” and “shares” are qualitatively different, involving different levels of effort, and endorsement or enjoyment of content. A further limitation of our study is our use of the Stewart and Furse [31] codebook that was developed to assess the use of television execution techniques on sales effectiveness. Here, we cannot comment on the relationship between sales effectiveness and social media engagement, or if such a relationship does exist. Also, our sample of posts covered a specific period (December to February), and we cannot comment on whether our findings are generalizable to alternative three-month periods. The observational design used here means that differences in engagement on each platform due to user demographics cannot be explored. It should also be noted that users of social media are not necessarily representative of the broader population and our findings must be generalized with caution. Further research examining qualitative aspects (eg, content analysis of comments) may provide further useful insights for future posts, as would detailed examination of the creative elements used to advertise different models of wearable devices (which are likely to offer varying features and benefits to users). Our results indicate that brands can leverage user participation by encouraging “sharing” of content, although to a much lesser extent than engaging through one-click “likes” or writing comments, future research should seek to explore this to understand how to increase user input to maximize engagement. Also, it is important to acknowledge that engagement with social media posts does not necessarily reflect real-life behavior such as purchasing of wearable products, or adherence to the healthy lifestyle elements promoted in the Fitbit and Garmin posts.

### Implications

This research offers several insights that may be useful for researchers developing social media-based health promotion campaigns and interventions in the future. First, it appears that Instagram is a promising platform for health promotion. In the literature to date, most efforts have been focused on Facebook, presumably as one of the earliest platforms with the largest user base. However, our study and background literature review suggest that Instagram achieves better reach to its audience, and vastly better engagement, highlighting the promise of this platform into the future. Key creative elements associated with highest “engagement” were the use of self-improvement themes, featuring “new” products and featuring the product in the image. Nonetheless, unlike wearables, most health promotion efforts do not have a concrete product to promote. However, intangible notions of improved health can be represented by tangibles, for example by focusing on the endpoint of related health benefits such as improved mood, vitality, and sleep quality, rather than the process of achieving these benefits. This finding also suggests that simple, clear, and direct messages may be best suited to social media. Just as wearable brands frequently post details of their new products and new features of their products, health promotion efforts should seek to refresh, rotate, and renew their health messages on a regular basis to attract engagement from social media users. Finally, differences in the Fitbit and Garmin approach underscore the need for health promotion efforts to clearly define their target population segment and tailor the messaging toward them. Clear examples of this from our study were targeting of gender, setting (indoors, urban, wilderness), and use of celebrities. That being said, there is evidence to suggest that people who engage with brands on social media are often existing users of the brand [33] and future work is required to determine how much social media engagement does reflect past or predict future behavior. Appealing content is more likely to receive attention and engagement from study participants. Several studies [6-8] have now demonstrated that higher engagement with the online component of an intervention translates to greater adherence to the behavior change aspects of the intervention, and is related to efficacious outcomes. This study offers important insights for researchers that will aid in the development of social media-based health promotion and intervention efforts that are appealing, and are therefore able to maximize the potential of these approaches.

### Conclusion

This study examined the social media activity of two wearable brands across three key social media platforms and provided novel insights into enhancing engagement. Future work should consider Instagram as a delivery platform and incorporate principles of market segmentation, or tailoring. Health messages on social media should be clear, direct, refreshed regularly, incorporate inspirational messages and imagery and be focused on tangible end products of health in order to maximize engagement and therefore the potential of this approach for positive behavior change.

## Appendix

Multimedia Appendix 1 Creative elements and descriptions.

Multimedia Appendix 2 Frequency of each creative element and comparison of use between brands.

## Abbreviations

AIC: Akaike information criterion
AIDA: awareness-interest-desire-action
BIC: Bayesian information criterion
eHealth: electronic health
mHealth: mobile health
