# Supplementary material for: Creating Engaging Health Promotion Campaigns on Social Media: Observations and Lessons From Fitbit and Garmin
Source: J Med Internet Res. 2018 Dec 10;20(12):e10911. doi: 10.2196/10911 (PMC6305879; doi:10.2196/10911)
Supplement: Multimedia Appendix 1 [file jmir_v20i12e10911_app1.pdf]

Supplementary Table 1. Creative elements and descriptions

Message or imagery feature	Description or example
Quality	May refer to craftsmanship or the quality of the product.
Aesthetic claims (styling, colour)	Relates to the appearance, style, fashion or of wearing the product.
Components or contents	For example, ‘counts steps’, ‘waterproof’, ‘join our app’, or shows someone using a specific device feature (e.g., checking step count).
Food or nutrition theme	For example, a link to a recipe (‘brighten up your salad routine with this refreshing recipe’), promotes benefits of diet (‘train like a pro, eat like a trainer’, ‘is red wine actually healthy’).
Exercise, fitness or physical activity theme	For example, suggests a workout (e.g., ‘here’s how to master 5 basic lunge moves’) or shows someone running.
Incidental activity theme	Related to incorporating more activity into day to day life (e.g., ‘take the stairs’).
Weight loss theme	Related to weight loss (e.g., ‘5 steps to crush your weight loss goals’ or ‘simple swaps to save calories’).
Sleep theme	For example, tips on to get a better night’s sleep or how to use the tracker sleep function.
Research findings	Gives a reason for using the product, ‘the research is in! 30 minutes of physical activity per day can…’, or relating to the product, ‘the science behind monitoring your activity’.
Special offer or event	Promotes a special offer or events such as sales, contests, two for one deals, premiums, limited time offers, or sponsorship of a race.
New or improved product features	Introduces a new or improved product (e.g., ‘now with constant heart rate monitor’, ‘longer battery life’, ‘slimmest band ever’).
Product advertised in image	Clearly visible in the image.
Product advertised in text	Mentioned in the text.
Scenic beauty	Scene of natural beauty (e.g., mountains, or flowing streams), does not have to be the primary focus of the post.
Text over image	Background image with text (e.g., inspirational quote) overlaid.
Product is the main focus	Focus is on displaying the product.
Social approval	Focus on winning friends, obtaining the approval of others or on using the device together with friends. May be explicit or implied.
Achievement	For example, winning a race, completing a marathon, winning a fishing competition or weight loss.
Self-improvement	Focus is on feeling better about oneself, self-improvement (health/fitness or related to emotions etc.) and setting personal

	goals.
Excitement, sensation or variety	Adding excitement, thrills, variety to life, or avoiding boredom. May include images of extreme sports.
Humorous	Funny, light-hearted amusement, comic timing, a sense of humour.
Rough and rugged	Relates to being tough, strong, capable, or enduring.
Puffery, or unsubstantiated claim	Product is declared best, better, or finest without identifying the dimension or attribute that is superior (e.g., ‘simply the best’, ‘when you want the best choose...’).
User experience	User experience of the product (e.g., before and after weight loss photos, training experience). Does not have to be explicit.
Camera involves audience	The camera is used to portray audience participation in the image (e.g., framed as users own eyes (looking at your own wrist with activity tracker on it) or as a close-up encounter (looking over someone's shoulder or very close to the image subject)).
Setting	No setting (e.g., coloured backdrop), indoor setting (e.g., office, kitchen), outdoor city or industrial setting (e.g., highway, footpath), outdoor nature setting (e.g., backyard, garden), wilderness setting (e.g., mountains, rivers).
Emotional or rational appeal	Emotional appeal creates a desire for the product without articulating specific features or benefits. Rational appeal creates a desire for the product by highlighting product attributes of benefits of ownership.
Negative or positive message frame	Negative frame (e.g., 'you won't regret getting moving', ‘won't win the race'). Positive frame (e.g., 'will help you stick to your goals', 'will remind you to move').
People	Number of people present in the image.
Children	Number of children present in the image.
Gender	Female, male, or males and females.
Racial or ethnic minorities present	Ethnicities other than Caucasian are present in the image.
Celebrity	A reasonably well known celebrity, actor, musician, athlete, Instagram-celebrity of well-known corporate figures.
Animal	An animal is present in the image.
