# Supplementary material for: Creating Engaging Health Promotion Campaigns on Social Media: Observations and Lessons From Fitbit and Garmin
Source: J Med Internet Res. 2018 Dec 10;20(12):e10911. doi: 10.2196/10911 (PMC6305879; doi:10.2196/10911)
Supplement: Multimedia Appendix 2 [file jmir_v20i12e10911_app2.pdf]

Supplementary Table 2. Frequency of each creative element and comparison of use between brands

Message or imagery feature	Total	Fitbit	Garmin	Chi-square test of homogeneity		
	Frequen	Frequen	Frequen	X2	df	p
Aesthetic claims	49 (9.6)	36 (13)	13 (5.6)	8.09	1	.004
Food or nutrition	53 (10.4)	51 (18.5)	2 (0.9)	42.05	1	.000
Exercise or physical activity	218	144	74 (31.8)	21.50	1	.000
Incidental activity	48 (9.4)	43 (15.6)	5 (2.1)	26.70	1	.000
Weight loss	22 (4.3)	22 (8)	0 (0)	19.41	1	.000
Sleep theme	10 (2)	10 (3.6)	0 (0)	8.61	1	.003
Components or contents	169	102 (37)	67 (28.8)	3.83	1	.05
Social approval	54 (10.6)	43 (15.6)	11 (4.7)	15.71	1	.000
Self-improvement	193	137	56 (24)	35.18	1	.000
New or improved	79 (15.5)	29 (10.5)	50 (21.5)	11.56	1	.001
Product text	236	99 (35.9)	137	27.71	1	.000
Scenic	117 (23)	18 (6.5)	99 (42.5)	92.33	1	.000
Excitement or variety	125	23 (8.3)	102	85.67	1	.000
Rough and rugged	90 (17.7)	9 (3.3)	81 (34.8)	86.16	1	.000
Children	10 (2)	0 (0)	10 (4.3)	12.08	1	.001
Celebrity	86 (16.9)	18 (6.5)	68 (29.2)	46.21	1	.000
Animal	19 (3.7)	0 (0)	19 (8.2)	23.38	1	.000
Quality	10 (2)	6 (2.2)	4 (1.7)	.14	1	.711
Setting				139.93	4	.000
Outdoor wilderness setting	102 (20)	10 (3.6)	92 (39.5)			
Outdoor nature setting	49 (9.6)	19 (16.9)	30 (12.9)			
Outdoor cityscape	81 (15.9)	47 (17)	34 (14.6)			
Indoor setting	159	132	27 (11.6)			
No setting	118	68 (24.6)	50 (21.5)			
Emotional or rational appeal				19.70	2	.000
Emotional appeal	165	71 (25.7)	94 (40.3)			
Rational appeal	218	142	76 (32.6)			
No appeal	126	63 (22.8)	63 (27)			
Negative or positive frame				77.64	2	.000
Negative frame	22 (4.3)	18 (6.5)	4 (1.7)			
Positive frame	282	195	87 (37.3)			
No frame	205	63 (22.8)	142			
Ethnicity				19.88	2	.000
Present	46 (9)	35 (12.7)	11 (4.7)			
Unsure	37 (7.3)	10 (3.6)	27 (11.6)			
Absent	426	231	195			
Gender				49.39	3	.000
Female	160	115	45 (19.3)			
Male	141	45 (16.3)	96 (41.2)			
Male and female	54 (10.6)	33 (12)	21 (9)			
No people	154	83 (30.1)	71 (30.5)			
Text over image	58 (11.4)	39 (14.1)	19 (8.2)	4.47	1	.35
Research findings	7 (1.4)	5 (1.8)	2 (0.9)	.85	1	.358
Special offer or event	100	48 (17.4)	52 (22.3)	1.94	1	.163
Product image	281	151	130	.06	1	.806
Product main focus	203	109	94 (40.3)	.04	1	.845
Achievement	54 (10.6)	29 (10.5)	25 (10.7)	.01	1	.935
Humorous	29 (5.7)	14 (5.1)	15 (6.4)	.44	1	.508
Puffery	8 (1.6)	4 (1.4)	4 (1.7)	.06	1	.809
User experience	46 (9)	22 (8)	24 (10.3)	.83	1	.361
Adult	352	193	159	.07	1	.681
Audience in image				1.36	2	.507
Close up image	125	65 (23.6)	60 (25.8)			
Image view through own eyes	82 (16.1)	41 (14.9)	41 (17.6)			
Absent	302	170	132			
